# Profiling of Phenolic Compounds and Antioxidant Activity of Dry Extracts from the Selected *Sorbus* Species

**DOI:** 10.3390/molecules17033093

**Published:** 2012-03-12

**Authors:** Monika A. Olszewska, Anna Presler, Piotr Michel

**Affiliations:** Department of Pharmacognosy, Faculty of Pharmacy, Medical University of Lodz, Muszyńskiego 1, 90‑151 Lodz, Poland; Email: anpre@wp.pl (A.P.); piotr.michel@umed.lodz.pl (P.M.)

**Keywords:** *Sorbus*, antioxidant activity, scavenging capacity, reducing power, linoleic acid peroxidation, DPPH, FRAP, TEAC, phenolic content, HPLC

## Abstract

The antioxidant efficiency of dry extracts from inflorescences and/or leaves of seven *Sorbus* species was studied using four *in vitro* tests of SET (single electron transfer) and HAT-type (hydrogen atom transfer) mechanisms. The 70% methanol extracts and its diethyl ether, ethyl acetate, *n*-butanol and water fractions were tested in parallel with the phenolic standards, e.g., caffeic acid, quercetin, BHA, BHT, and Trolox^®^. The SET-type activity of the extracts depended primarily on the extraction solvent. The most valuable extracts were *n*-butanol and ethyl acetate ones, which activity was high in the DPPH (EC_50_ = 3.2–5.2 μg/mL), TEAC (2.8–4.0 mmol Trolox^®^/g), and FRAP (9.8–13.7 mmol Fe^2+^/g) tests, and strongly correlated with the total phenolic levels (39.6–58.2% of gallic acid equivalents). The HPLC-PDA analysis of the extracts led to the identification of chlorogenic acid, isoquercitrin, hyperoside, rutin, quercetin 3-*O*-sophoroside, and sexangularetin 3-*O*-*β*-D-glucopyranoside as the main components. Apart from flavonoids and hydroxycinnamic acids, proanthocyanidins have also a significant impact on the SET-type activity. The HAT-reactivity of the extracts in the linoleic acid peroxidation test (IC_50_ = 36.9–228.3 μg/mL) depended more strongly on the plant tissue than on the extraction solvent, and its correlation with the phenolic content was weak. Both SET and HAT-type activity of the most potent *Sorbus* extracts was comparable with the activity of the standards, indicating their great potential as effective sources for health products.

## 1. Introduction

Plants constitute an important source of potent natural antioxidants, which differ widely in terms of chemical structure and biological properties. The most important group of plant antioxidants are phenolics, which are recognised as beneficial to human health, mostly due to their ability to neutralise reactive oxygen species (ROS) [1,2,3]. ROS, including free radicals, are generated in physiological reactions of normal human metabolism or in the presence of various environmental stressors [2]. If not properly regulated by the endogenous defence system, ROS can react with important biomolecules, causing cellular injury, accelerated aging and the development of chronic diseases, such as atherosclerosis, coronary diseases, cancer, and neurodegenerative brain disorders [1,2]. The protective effect of the internal antioxidant system can be significantly enhanced by exogenous antioxidants, including plant phenolics that are supplied to humans as food components or as specific preventive pharmaceuticals [3]. Endogenous and exogenous antioxidants act interactively to maintain or re-establish redox homeostasis, which is critical in maintaining a healthy biological system [2]. Many phenolic constituents of herbal medicines and dietary plants have been identified as safe and potent exogenous antioxidants, and the antioxidant effectiveness of plant extracts is suggested as a superior alternative for the single phenolic compounds, both natural and synthetic, due to the synergistic action of a wide range of active molecules existing in plant products [3]. Moreover, supplementation with isolated, pure compounds outside of their natural matrix can lead to the overdose of antioxidants, resulting in disruption of cellular redox balance and pro-oxidant effects [2]. The excessive use of synthetic antioxidants, such as BHA or BHT, is also burdened with the risk of toxic and carcinogenic effects [2,3]. Accordingly, there is still a growing interest in finding natural materials and plant extracts exhibiting sufficiently potent activity to effectively replace the synthetic compounds.

The genus *Sorbus sensu lato* (Rosaceae, Maloideae) is represented by about 250 species of trees and shrubs, being commonly found throughout the Northern Hemisphere. As treated in its broad sense, the genus is taxonomically divided into four subgenera (*Aria*, *Cormus*, *Sorbus* and *Torminaria*). The major subgenus *Sorbus*, otherwise known as the genus *Sorbus sensu stricto*, includes only the pinnate leaved species grouped around the model *Sorbus aucuparia* L. [4]. Various *Sorbus* taxa have been traditionally used for ethnomedical properties, such as anti-diarrhoeal, diuretic, anti-inflammatory, anti-diabetic, vasoprotective, broncho- and vasorelaxant activities, and they are also known to be potent antioxidant agents [5,6,7,8,9]. In the course of our continuing studies of antioxidants in *Sorbus*, the plant materials derived from the *Sorbus s.s.* species have been found to exhibit higher antioxidant activity than those obtained from representatives of other subgenera [7,8,9], and this activity has been attributed to the high phenolic content. Statistical cluster analysis of the screening data identified the ten tissues, e.g., inflorescences of *S. aucuparia*, exhibiting the greatest potential as effective sources for natural health products [8]. However, the previous investigations have been conducted with the use of liquid 70% methanol extracts prepared *in situ* from small analytical samples. Further study of the semi-preparative-scale extraction efficiency, direct comparison between the activity of dry extracts and the most popular commercial antioxidants and profiling of individual native phenolics is required to fully characterise the *Sorbus* plant materials as antioxidant remedies. Analysis of powdered extracts is very important, since in this form natural antioxidants can be long-term stored before the use as food or pharmaceutical additives.

Therefore, the aim of this project was to investigate the extraction efficiency and the antioxidant capacity of the dry lipophilic (chloroform) and polar (70% methanolic) extracts and its various solvent fractions obtained from eight tissues of the selected *Sorbus* s.s. species, which have been found previously [8] to possess the highest phenolic content. The activity of the extracts was studied using four *in vitro* test systems of complementary mechanisms *versus* the most popular natural and synthetic standard antioxidants. The phenolic profiles of the extracts were extensively studied by spectrophotometric and HPLC-PDA fingerprint methods. Moreover, the impact of the extraction solvent and the phenolic level on the antioxidant activity of the extracts was investigated statistically.

## 2. Results and Discussion

### 2.1. Semi-Preparative Extraction of the Sorbus Tissues

The extraction yield obtained from the *Sorbus* tissues on a semi-preparative scale is reported in Table 1. The yield of hydrophilic components extractable with 70% methanol (ME) varied from 25.9% to 32.3% (w/w) of the dry plant material (dw), depending on the plant sample tested, and it was higher than that of lipophilic fractions extractable with chloroform (CHE) and ranging between 3.5–9.0% dw. Among the organic solvents used for fractionation of ME, the highest extraction efficiency (5.1–8.0% dw) was observed for *n*-butanol (BF), followed by that of ethyl acetate (EAF, 0.6–2.6% dw) and diethyl ether (DEF, 0.2–0.6% dw).

**Table 1:** .Extraction efficiency of the analysed *Sorbus* dry extracts and fractions.

Sample No.	Plant source		Extraction yield (% dw) *^b^*					
	Scientific name	Plant part tested *^a^*	CHE	ME	DEF	EAF	BF	WR
1.	*Sorbus aucuparia* L.	I	3.5	32.3	0.4	2.2	6.3	23.4
2.	*Sorbus commixta* Hedl.	I	6.1	26.3	0.6	2.6	6.1	17.0
3.	*Sorbus decora*(Sarg.) C.K. Schneid.	I	4.6	31.9	0.3	2.5	5.5	23.2
4.	*Sorbus gracilis* (Sieb. & Zucc.) K. Koch	I	4.3	28.9	0.4	0.6	7.1	19.8
5.	*Sorbus gracilis* (Sieb. & Zucc.) K. Koch	L	6.2	25.9	0.3	1.4	6.5	15.8
6.	*Sorbus koehneana* C.K. Schneid.	I	5.6	30.4	0.3	0.9	5.7	21.6
7.	*Sorbus pogonopetala* Koehne	L	8.8	30.8	0.2	1.0	5.1	21.9
8.	*Sorbus wilfordii* Koehne	L	9.0	29.6	0.2	1.0	8.0	18.5

*^a^* I, inflorescence; L, leaf. *^b^* Extraction yield calculated for dry weight of the plant material. Codification of the extracts and fractions: CHE, chloroform extract; ME, 70% methanol extract; DEF, diethyl ether fraction; EAF, ethyl acetate fraction; BF, *n*-butanol fraction; WR, water residue.

### 2.2. Total Phenolic Content and SET-Type Antioxidant Activity of the Sorbus Dry Extracts *versus* Phenolic Standards

In our previous work [8] it was proved that the total phenolic content (TPC) as determined by the Folin-Ciocalteu (FC) assay is a good approximate of the total level of the main phenolic metabolites of *Sorbus* tissues, including flavonoids, proanthocyanidins and caffeoylquinic acids. Thus, the FC method was chosen in the present study to screen the phenolic content of the analysed samples (Table 2).

**Table 2:** Total phenolic content and SET-type antioxidant activity of the analysed *Sorbus* dry extracts and fractions *^a^*.

Sample No.	Extract/ Fraction	Total phenolic content (TPC) *^b^*	Radical-scavenging activity (RSC) *^c^*		Reducing power *^d^*
		GAE (%)	DPPH EC_50 _(µg/mL)	TEAC (mmol Trolox^®^/g)	FRAP (mmol Fe^2+/^g)
1.	ME	21.17 ± 0.67 *^M^*	8.93 ± 0.27 *^I^*	1.72 ± 0.06 *^L^*	4.43 ± 0.14 *^N,P^*
	DEF	37.61 ± 0.37 *^H,I^*	5.53 ± 0.22 *^E,F^*	2.14 ± 0.09 *^I,J,K^*	9.30 ± 0.38 *^G,H,I^*
	EAF	54.34 ± 0.46 *^B,C^*	3.37 ± 0.18 *^A,B,C^*	3.22 ± 0.10 *^E^*	12.77 ± 0.12 *^B,C^*
	BF	48.71 ± 1.27 *^E,F^*	3.52 ± 0.13 *^A,B,C^*	3.58 ± 0.12 *^C,D^*	10.84 ± 0.17 *^F^*
	WR	9.05 ± 0.15 *^P^*	9.96 ± 0.19 *^K^*	0.94 ± 0.04 *^R^*	2.58 ± 0.05 *^R,S^*
2.	ME	23.77 ± 0.30^*L,M*^	7.16 ± 0.22 *^G^*	1.70 ± 0.10 *^L^*	5.04 ± 0.24 *^M,N^*
	DEF	36.67 ± 0.49 *^H I^*	5.72 ± 0.20 *^E,F^*	2.14 ± 0.05 *^I,J,K^*	7.58 ± 0.10 *^K^*
	EAF	53.55 ± 1.13 *^C,D^*	3.52 ± 0.13 *^A,B,C^*	2.62 ± 0.13 *^H^*	12.23 ± 0.07 *^C,D^*
	BF	48.52 ± 0.53 *^E,F,G^*	3.53 ± 0.16 *^ A,B,C^*	3.40 ± 0.08 *^D,E^*	11.01 ± 0.59 *^E,F^*
	WR	11.00 ± 0.12 *^P^*	9.66 ± 0.25 *^K^*	1.26 ± 0.05 *^N^*	2.70 ± 0.13 *^R,S^*
3.	ME	24.61 ± 0.82 *^ L^*	7.76 ± 0.16 *^H^*	1.79 ± 0.09 *^L^*	5.42 ± 0.16 *^M^*
	DEF	34.50 ± 0.89 *^I,J^*	5.57 ± 0.14 *^E,F^*	2.67 ± 0.08 *^G,H^*	8.50 ± 0.10 *^J^*
	EAF	55.16 ± 0.79 *^A,B,C^*	3.44 ± 0.07 *^A,B,C^*	3.98 ± 0.14 *^A^*	13.74 ± 0.16 *^A^*
	BF	53.75 ± 1.62 *^B,C,D^*	3.17 ± 0.11 *^A^*	3.55 ± 0.11 *^B,C,D^*	11.47 ± 0.11 *^E,F^*
	WR	10.06 ± 0.66 *^P^*	9.84 ± 0.19 *^K^*	1.21 ± 0.03 *^N^*	2.77 ± 0.05 *^R,S^*
4.	ME	24.63 ± 0.22 *^L^*	7.93 ± 0.16 *^H^*	1.99 ± 0.04 *^K^*	5.36 ± 0.28 *^M^*
	DEF	36.87 ± 0.80 *^H,I^*	5.39 ± 0.21 *^D,E^*	2.71 ± 0.07 *^G,H^*	9.34 ± 0.30 *^G,H^*
	EAF	54.09 ± 0.34 *^B,C^*	3.71 ± 0.18 *^B,C^*	3.65 ± 0.12 *^B,C^*	13.06 ± 0.26 *^B^*
	BF	57.09 ± 0.50 *^A,B^*	3.25 ± 0.12 *^A,B^*	3.68 ± 0.12 *^B,C^*	9.92 ± 0.36 *^G^*
	WR	8.21 ± 0.31 *^P^*	10.12 ± 0.21 *^K^*	1.15 ± 0.04 *^N,P^*	2.26 ± 0.06 *^S^*
5.	ME	30.62 ± 0.60 *^K^*	6.60 ± 0.14 *^G^*	2.12 ± 0.08 *^I,J,K^*	6.20 ± 0.25 *^L^*
	DEF	34.90 ± 0.27 *^I,J^*	5.29 ± 0.18 *^D,E^*	2.14 ± 0.07 *^I,J,K^*	8.72 ± 0.27 *^H,I,J^*
	EAF	52.37 ± 0.38 *^C,D^*	3.70 ± 0.08 *^B,C^*	3.72 ± 0.12 *^B^*	12.94 ± 0.30 *^B^*
	BF	48.62 ± 1.02 *^F,G^*	3.83 ± 0.17 *^C^*	3.33 ± 0.10 *^E^*	11.05 ± 0.35 *^E,F^*
	WR	11.45 ± 0.28 *^P^*	9.54 ± 0.21 *^J,K^*	1.31 ± 0.05 *^N^*	2.98 ± 0.11 *^R^*
6.	ME	26.38 ± 0.91 *^L^*	6.74 ± 0.13 *^G^*	2.08 ± 0.10 *^J,K^*	5.44 ± 0.25 *^M^*
	DEF	32.10 ± 0.33 *^J,K^*	5.70 ± 0.12 *^E,F^*	2.60 ± 0.10 *^H^*	8.38 ± 0.23 *^J^*
	EAF	50.51 ± 0.95 *^D,E^*	3.46 ± 0.17 *^A,B,C^*	3.56 ± 0.13 *^C,D^*	12.87 ± 0.17 *^B^*
	BF	58.17 ± 0.76 *^A^*	3.15 ± 0.13 *^A^*	3.94 ± 0.15 *^A^*	9.81 ± 0.19 *^G^*
	WR	10.51 ± 0.30 *^P^*	9.71 ± 0.22 *^K^*	1.29 ± 0.04 *^N^*	2.54 ± 0.15 *^R,S^*
7.	ME	24.03 ± 0.23 *^L,M^*	6.84 ± 0.16 *^G^*	1.81 ± 0.09 *^L^*	5.54 ± 0.20 *^M^*
	DEF	42.85 ± 0.87 *^G^*	4.89 ± 0.14 *^D^*	2.28 ± 0.08 *^I^*	10.92 ± 0.11 *^F^*
	EAF	53.29 ± 0.23 *^C,D^*	3.80 ± 0.14 *^C^*	3.44 ± 0.10 *^D,E^*	11.42 ± 0.47 *^E,F^*
	BF	39.56 ± 1.47 *^H^*	5.18 ± 0.11 *^D,E^*	2.96 ± 0.12 *^F^*	8.67 ± 0.22 *^I,J^*
	WR	10.38 ± 0.51 *^P^*	9.83 ± 0.27 *^K^*	1.03 ± 0.04 *^P,R^*	2.92 ± 0.04 *^R^*
8.	ME	29.93 ± 0.43 *^K^*	6.01 ± 0.23 *^F^*	2.24 ± 0.11 *^I,J^*	6.78 ± 0.16 *^L^*
	DEF	53.13 ± 1.38 *^C,D^*	3.67 ± 0.13 *^B,C^*	2.97 ± 0.12 *^F^*	11.60 ± 0.15 *^D,E^*
	EAF	54.34 ± 0.32 *^B,C^*	3.45 ± 0.16 *^A,B,C^*	3.41 ± 0.11 *^D,E^*	12.55 ± 0.54 *^B,C^*
	BF	48.37 ± 0.51 *^E,F,G^*	3.28 ± 0.15 *^A,B^*	2.83 ± 0.11 *^G^*	10.99 ± 0.09 *^E,F^*
	WR	15.27 ± 0.18 *^N^*	9.04 ± 0.26 *^I,J^*	1.51 ± 0.08 *^M^*	4.03 ± 0.11 *^P^*

*^a^* Results are mean values of replicate analyses (*n* = 2 × 5 × 1) ± SD calculated per dry weight of the extract or fraction. Different superscripts (capitals) in each column indicate significant differences in the mean values at *p* < 0.01. Codification of the samples, extracts and fractions is given in Table 1. *^b^* Total phenolic content expressed in GAE, gallic acid equivalents. *^c^* Scavenging efficiency (EC_50_, effective concentration, amount of antioxidant needed to decrease the initial DPPH concentration or the initial absorbance of the ABTS solution by 50%) expressed in µg/mL for the DPPH test or in TEAC, millimolar Trolox^®^ antioxidant equivalents/g for the ABTS assay. *^d^* Ferric reducing antioxidant power.

**Figure 1:**
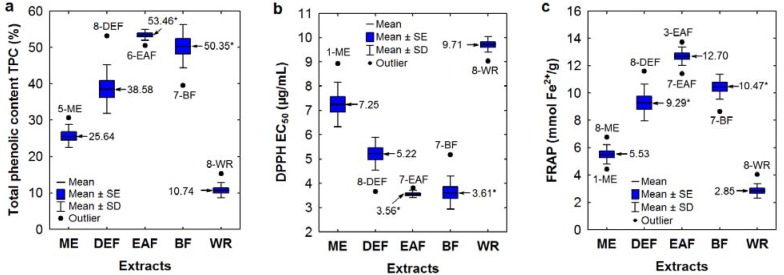
(**a**–**c**) Variation in total phenolic levels TPC and SET-type antioxidant activity among the *Sorbus* extracts depending on the type of extraction solvent. Sample codes are given acc. to Table 2. Mean values are given ± standard error (SE) and standard deviation (SD). Mean values marked with an asterisk are not significantlydifferent (*p* < 0.01).

The measured TPC levels, expressed as gallic acid equivalents (GAE), were affected primarily by the extracting solvents as shown in the boxplot (Figure 1a). The highest TPC contents were observed for EAFs and BFs (48.52–58.17% dw of the extract) with one outlier found for BF of the *S. pogonopetala* leaf (39.56%), and with no significant differences between the average values for both extract groups. A similar TPC level was also noted in DEF of the *S.* *wilfordii* leaf (53.13%). Extremely low TPC content was found for the CHEs (0.71–0.75%, results not shown), which were thus excluded from the activity and HPLC fingerprint studies.

**Figure 2:**
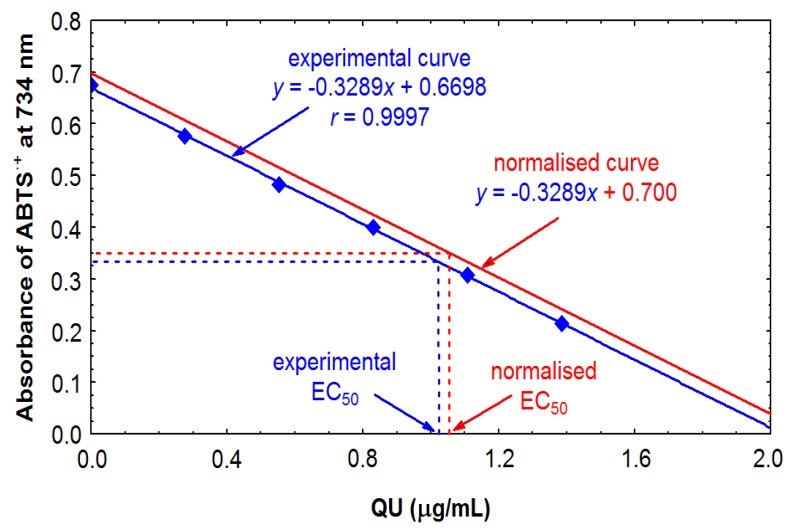
Scavenging of the ABTS radical cation by quercetin (QU)–example of normalisation of EC_50_ value with the absorbance of 0.700.

The free radical scavenging activity (RSC) of the analytes was tested by two discolouration methods, such as the DPPH [10] and ABTS (TEAC III) [11] assays. In these methods, the antiradical capacity is expressed as the percentage decrease of the initial concentration of the DPPH radical or the initial absorbance of the ABTS^●^^+^ solution, and further characterised by the EC_50_ values. Since the results are strongly affected by the initial parameters, constant reaction conditions are crucial to maintain accuracy. The common practice to equilibrate the radical solutions to the initial absorbance of 0.700 ± 0.020 (0.030) [7,8,9,10,12] is only partially effective, because even small differences in the initial absorbance could lead to scattered values of EC_50_. Moreover, the ABTS^●^^+^ radical cation is very unstable [13] and the DPPH solution is sensitive to light [15], thus both reagents could slowly deteriorate during the reaction period. On the other hand, the ratio between the initial DPPH concentration and the EC_50_ value is constant [15]. Therefore, in the present work we proposed to enhance the accuracy of the scavenging tests by the following procedure: once the initial absorbances were equilibrated, the negative controls were incubated simultaneously with the real samples to compensate possible deterioration of the radical reagents, and the calculated original values of EC_50_ were normalised with the constant initial parameters (DPPH concentration of 25 μg/mL and absorbance of the ABTS^●^^+^ solution of 0.700) by simple mathematic conversions (see Section 3.5 and Section 3.6). A graphical example of normalisation of the ABTS test is shown in Figure 2.

The normalised EC_50_ and TEAC values of the *Sorbus* extracts varied from 3.15 to 10.12 μg/mL for the DPPH test, and from 0.94 to 3.98 mmol Trolox^®^/g for the TEAC assay (Table 2). High consistency of the RSC values determined by the two methods was confirmed by a statistically significant linear correlation (*r* = −0.9258, *p* < 0.01). The activity parameters of the dry extracts were also significantly (*p* < 0.01) correlated with the TPC content. The correlation was strong for both methods, DPPH (*r* = −0.9850) and TEAC (*r* = 0.9361). For the DPPH tests the correlation with the TPC levels was even stronger than found previously for the *in situ* methanolic liquid extracts [8], which could be a consequence of the purification and concentration of phenolics during the preparation of dry extracts.

Similarly as observed for the FC method, variation in the RSC values for both antiradical tests was primarily caused by the differences in extraction solvents (Figure 1b), and the highest activity was found for EAFs and BFs with no significant differences (*p* < 0.01) between the average RSC values for these extract types. In the DPPH test, the EC_50_ values of EAFs and BFs varied in a narrow range of 3.15–3.83 μg/mL with one outlier for BF of *S. pogonopetala* leaf (5.18 μg/mL). The range of the TEAC values was slightly wider (2.83–3.98 mmol Trolox^®^/g, including outliers). This activity was comparable or even higher than the RSC of the phenolic standards, such as BHA, BHT, CHA, RT, TBHQ and Trolox^®^ (Table 3). Activity of CA, CFA, GA and QU was 2–3 times higher. Differences in RSC values between *Sorbus* extracts and standards were more pronounced in the TEAC assay, and they could be explained by different steric accessibility of the radical sites of ABTS^●^^+^ and DPPH radicals to small molecular standards and larger molecules [13], such as *Sorbus* phenolics including proanthocyanidins, flavonoid glycosides and caffeoylquinic acids (see Section 2.3).

**Table 3:** Antioxidant activity of the reference standards *^ a^*.

Standard *^b^*	Radical-scavenging activity (RSC) *^c^*		Reducing power *^d^*	LA peroxidation *^e^*
	DPPH EC_50_ (µg/mL)	TEAC (mmol Trolox^®^/g)	FRAP (mmol Fe^2+^/g)	IC_50_ (µg/mL)
CFA	1.94 ± 0.08 *^A^*	10.37 ± 0.17 *^C^*	44.17 ± 0.98 *^A^*	24.96 ± 1.34 *^C^*
CA	2.17 ± 0.11 *^A^*	9.51 ± 0.53 *^D^*	25.37 ± 0.44 *^C^*	69.68 ± 0.70 *^G^*
CHA	4.42 ± 0.13 *^C^*	4.13 ± 0.10 *^G^*	18.04 ± 0.79 *^D^*	52.47 ± 2.03 *^F^*
GA	0.95 ± 0.05 *^D^*	22.36 ± 0.63 *^A^*	43.52 ± 1.93 *^A^*	23.97 ± 0.98 *^B,C^*
QU	1.63 ± 0.07 *^E^*	12.41 ± 0.11 *^B^*	36.02 ± 1.10 *^B^*	48.51 ± 1.74 *^E^*
RT	3.44 ± 0.09 *^F^*	4.45 ± 0.15 *^G^*	11.89 ± 0.70 *^F^*	67.73 ± 0.34 *^G^*
BHA	2.90 ± 0.14 *^B^*	7.09 ± 0.17 *^E^*	16.13 ± 0.83 *^E^*	14.33 ± 0.70 *^A^*
BHT	6.54 ± 0.28 *^G^*	2.56 ± 0.08 *^H^*	18.89 ± 0.42 *^D^*	21.58 ± 0.95 *^B^*
TBHQ	2.73 ± 0.12 *^B^*	6.01 ± 0.24 *^F^*	15.50 ± 0.71 *^E^*	36.53 ± 1.04 *^D^*
Trolox^®^	4.34 ± 0.22 *^C^*	3.99 ± 0.10 *^G^*	10.83 ± 0.32 *^F^*	22.45 ± 1.10 *^B,C^*

*^a^* Results are mean values of replicate analyses ± SD. Different superscripts (capitals) in each column indicate significant differences in the mean values at *p* < 0.01. *^b^* Codification of the standards: CFA, caffeic acid; CA, (+)-catechin; CHA, chlorogenic acid; GA, gallic acid; QU, quercetin; RT, rutin; BHA, butylated hydroxyanisole; BHT, butylated hydroxytoluene; TBHQ, *tert*-butylhydrochinon. *^c^* Scavenging efficiency (EC_50_, effective concentration, amount of antioxidant needed to decrease the initial DPPH concentration or the initial absorbance of the ABTS solution by 50%) expressed in µg/mL for the DPPH test and inmillimolar Trolox^®^ antioxidant equivalents (TEAC)/g for the ABTS assay. *^d^* Ferric reducing antioxidant power. *^e^* Inhibition of linoleic acid (LA) peroxidation (IC_50_, inhibition concentration, amount of antioxidant needed to decrease the LA peroxidation by 50%).

In the FRAP method, the antioxidant activity is determined based on the ability to reduce Fe^3+^ to Fe^2+^, and the results are expressed as millimolar ferrous ion equivalents per gram of the sample [16]. The FRAP values obtained for *Sorbus* extracts paralleled the TPC levels and the results of TEAC and DPPH tests (Table 2, Figure 1c). It is confirmed by a highly significant (*p* < 0.01) linear correlation found between the FRAP values and TPC levels (*r* = 0.9671), TEAC values (*r* = 0.9064), and EC_50_ values of the DPPH test (*r* = −0.9638). The highest FRAP values were observed for EAFs (11.42–13.74 mmol Fe^2+^/g, including outliers), BFs (9.81–11.47 mmol Fe^2+^/g, without the outlier for *S. pogonopetala* leaf), and DEFs (7.58–11.60 mmol Fe^2+^/g, without the outlier for *S. wilfordii* leaf). The FRAP activity of the most active extracts was comparable to the activity of RT and Trolox^®^, but it is 1.5–4 times lower than those of the other analysed standards (Table 3). For some small molecular phenolics, such as CFA, GA, and CA, their extremely high FRAP activity expressed in weight units was affected by low molecular mass. If expressed in molar units, FRAP values of these standards did not differ significantly (*p* < 0.01) from the activity of RT. The observed differences in FRAP activity between small molecular phenolics and *Sorbus* extracts abundant in macromolecular tannin-type proanthocyanidins (see Section 2.3) could also be explained by different reaction kinetics of reagents differing in molecular weight [16]. However, the slow reaction rate of plant extracts implies an ability to retain and even increase their reducing ability with time [13,16], and might thus signify a longer protecting effect against oxidative damage *in vivo*.

The determined SET-type antioxidant activity of standards (Table 3) was in accordance with the previous reports [11,16,17] in terms of overall order and magnitude, which validated the results obtained. Some slight discrepancies may be due to the differences in the reaction conditions, such as the initial reagent concentration and analysis run time, and also because of normalisation of the EC_50_ values, which was employed in the present study for DPPH and TEAC tests.

Relationships between SET-type antioxidant activity parameters of standards, although statistically significant (*p* < 0.05) and linear, were weaker than those of *Sorbus* extracts, which was evidenced by lower correlation coefficients for e.g., the DPPH and TEAC tests (*r* = −0.8109) or the TEAC and FRAP assays (*r* = 0.825). Higher *r-*values found for the *Sorbus* extracts indicated synergistic and additive effects of their antioxidant constituents. These effects have been documented for several other plant extracts containing phenolics, and can be explained by complementary reactivity and regeneration mechanisms between individual antioxidants, depending on their structures and on the possible formation of stable intermolecular complexes [20].

Direct comparison of our antioxidant results with the literature data is very difficult, given the varying assay protocols utilised by different authors. On the other hand, the TPC levels are easy to compare and can be considered as an indirect measure of antioxidant activity because of the basic redox mechanism and standardised conditions of the FC method. Among the natural products, the extracts of tea leaf and grape seed appear to have the greatest antioxidant potential. The highest TPC values have been reported for the commercial ethanol extract (EE) of grape seed (60% GAE [19]), EAFs of green tea (58% GAE [20]) and green mate (42–48% GAE [20]), and followed by those of 80–100% MEs (23–37% GAE [20,21]) obtained from the last two plant materials. There are only a few other plant extracts exhibiting comparable TPC levels, e.g., EE of *Magnifera indica* leaf (59–65% GAE [19]), ME of the *Hypericum foliosum* stem (39% GAE [22]) or *Syzygium aqueum* leaf (52% GAE [19]). In this context, the tested *Sorbus* extracts appear to be very rich sources of natural antioxidants (39–58% GAE in EAFs and BFs).

### 2.3. Phenolic Profile of the Analysed Sorbus Dry Extracts and Fractions

It is evident that the TPC value determined by the FC assay does not give a full picture of the real phenolic constituents in plant extracts. Thus, for verification of the phenolic levels in *Sorbus*, further determinations of the main phenolic groups were performed. Results of the appropriate HPLC-PDA and UV-spectrophotometric assays are reported in Table 4 and Table 5.

**Table 4:** Total content of proanthocyanidins, hydroxybenzoic acids and flavonoids in the analysed *Sorbus* dry extracts and fractions *^a^*.

Sample No. /Extract/ Fraction		Total proanthocyanidin content (%) *^b^*	Hydroxybenzoic acids (%) *^c^*	Flavonoids (%) *^d^*
1.	ME	11.16 ± 0.37 *^I,J^*	0.14 *^G,H^* (PCA: 0.05)	5.83 *^H^* (SQ: 0.70; HY: 0.95; IQ: 1.67; GS: 0.94)
	DEF	1.21 ± 0.05 *^A,B,C,D^*	2.69 *^R^* (PCA: 1.65; *p*HBA: 0.47)	7.92 *^K^* (HY: 1.39; IQ: 3.11; GS: 1.46; QU: 0.38)
	EAF	9.81 ± 0.16 *^G,H^*	0.10 (PCA: 0.10) *^D,E,F^*	36.22 *^T^* (HY: 9.02; IQ: 16.15; GS: 7.25)
	BF	36.08 ± 0.59 *^R^*	0.13 *^F,G^*	12.26 *^N^* (SQ: 3.55; RT: 2.22; IQ: 1.12)
	WR	1.95 ± 0.07 *^C,D,E^*	0.08 *^B,C,D^*	not detected
2.	ME	8.00 ± 0.36^*F*^	0.17 *^I^* (PCA: 0.05)	1.67 *^C^* (HY: 0.92; IQ: 0.22; GS: 0.18)
	DEF	0.57 ± 0.03 *^A,B^*	2.19 *^P^* (PCA: 1.41; *p*HBA: 0.74)	9.68 *^L^* (HY: 1.65; IQ: 0.54; GS: 0.49; QU: 1.04)
	EAF	8.01 ± 0.19 *^F^*	0.21 *^J^* (PCA: 0.15)	21.21 *^S^* (HY: 11.60; IQ: 2.95; GS: 2.41)
	BF	26.51 ± 0.61 *^Q^*	0.22 *^J,K^*	1.44 *^B,C^* (RT: 0.22; HY: 0.81; IQ: 0.16; GS: 0.15)
	WR	0.74 ± 0.04 *^A,B,C^*	0.14 *^G,H^*	not detected
3.	ME	10.22 ± 0.16 *^ H,I^*	0.07 *^B,C^* (PCA: 0.02)	3.47 *^D,E^*(RT: 0.61; HY: 0.87; IQ: 0.38; GS: 0.24)
	DEF	0.96 ± 0.08 *^A,B,C,D^*	2.20 *^P^*(PCA: 1.29; *p*HBA: 0.40)	5.87 *^H^* (HY: 1.66; IQ: 0.76; GS: 0.53; QU: 1.02)
	EAF	10.83 ± 0.09 *^H,I^*	0.06 *^A,B^*(PCA: 0.06)	20.15 *^R^* (HY: 10.67; IQ: 3.91; GS: 3.20)
	BF	38.36 ± 1.13 *^S^*	0.09 *^C,D,E^*	13.90 *^O^* (RT: 3.67; HY: 1.24; IQ: 0.78)
	WR	1.10 ± 0.02 *^A,B,C,D^*	0.06 *^A,B^*	0.08 *^A^*
4.	ME	17.64 ± 0.42 *^N,O^*	0.11 *^E,F,G^* (PCA: 0.06)	1.51 *^B,C^* (RT: 0.23; HY: 0.10; GS: 0.21)
	DEF	1.00 ± 0.04 *^A,B,C,D^*	3.32 *^S^* (PCA: 2.50; *p*HBA: 0.77)	1.92 *^C^* (HY: 0.31; IQ: 0.22; GS: 0.83; QU: 0.20)
	EAF	13.90 ± 0.25 *^L^*	0.22 *^J,K^* (PCA: 0.22)	8.12 *^K^* (RT: 0.55; HY: 1.29; IQ: 0.57; GS: 3.37)
	BF	46.11 ± 0.68 *^T^*	0.21 *^J^*	4.96 *^F,G^* (RT: 1.45; HY: 0.14)
	WR	2.13 ± 0.01 *^D,E^*	0.11 *^E,F^*	not detected
5.	ME	14.22 ± 0.32 *^L^*	0.06 *^A,B^* (PCA: 0.03)	3.85 *^E^* (SQ: 0.08; HY: 0.19)
	DEF	0.93 ± 0.07 *^A,B,C,D^*	0.56 *^L^* (PCA: 0.51; *p*HBA: 0.05)	4.61 *^F^* (QU: 0.19)
	EAF	17.13± 0.27 *^N^*	0.05 *^A,B^* (PCA: 0.05)	14.73 *^P^* (RT: 0.53)
	BF	39.04 ± 1.10 *^S^*	not detected	6.39 *^J^* (SQ: 0.28; HY: 0.45)
	WR	0.33 ± 0.03 *^A^*	not detected	0.53 *^A^*
6.	ME	16.81 ± 0.19 *^N^*	0.24 *^K^* (PCA: 0.06; *p*HBA: 0.03)	1.52 *^B,C^* (SQ: 0.50; RT: 0.47; HY: 0.18; GS: 0.20)
	DEF	1.26 ± 0.07 *^A,B,C,D^*	1.95 *^O^* (PCA: 0.92; *p*HBA: 1.03)	1.11 *^B^* (HY: 0.34; IQ: 0.46; QU: 0.16)
	EAF	15.53 ± 0.19 *^M^*	0.23 *^J,K^* (PCA: 0.13)	7.81 *^K^* (RT: 1.05; HY: 2.29; GS: 2.63)
	BF	51.20 ± 1.24 *^U^*	0.21 *^J^*	5.42 *^G,H^* (SQ: 2.64; RT: 2.21)
	WR	2.72 ± 0.08 *^E^*	0.15 *^H,I^*	not detected
7.	ME	8.56 ± 0.29 *^F,G^*	0.03 *^A^*	3.13 *^D^* (SQ: 0.57; RT: 0.22; HY: 0.17; IQ: 0.30)
	DEF	1.19 ± 0.11 *^G^*	1.20 *^N^* (PCA: 0.72; *p*HBA: 0.49)	8.04 *^K^* (IQ: 0.32; QU: 0.71)
	EAF	12.36 ± 0.11 *^J,K^*	0.07 *^B,C,D^* (PCA: 0.07)	5.03 *^F,G^* (HY: 0.90; IQ: 1.69; QU: 0.16)
	BF	20.55 ± 0.20 *^P^*	0.12 *^E,F,G^*	11.55 *^M^* (SQ: 3.00; RT: 1.15; IQ: 0.29)
	WR	0.25 ± 0.03 *^A^*	0.06 *^B,C^*	0.17 *^A^*
8.	ME	12.55 ± 0.31 *^K^*	not detected	5.55 *^H^* (SQ: 3.67; RT: 0.68)
	DEF	1.61 ± 0.05 *^B,C,D,E^*	0.89 *^M^* (PCA: 0.75; *p*HBA: 0.11)	3.42 *^D,E^* (QU: 0.27)
	EAF	18.62 ± 0.51 *^O^*	0.08 *^B,C,D^* (PCA: 0.08)	6.61 *^I^* (SQ: 0.67; RT: 0.53; IQ: 0.14)
	BF	26.81 ± 0.27 *^Q^*	not detected	18.15 *^Q^* (SQ: 13.37; RT: 2.50)
	WR	1.43 ± 0.03 *^A,B,C,D^*	not detected	0.36 *^A^* (SQ: 0.36)

*^a^* Results are mean values of replicate analyses calculated per dry weight of the extract or fraction. Different superscripts (capitals) in each column indicate significant differences in the mean values at *p* < 0.05. Codification of the samples, extracts and fractions is given in Table 1. *^b^* Total proanthocyanidin content expressed in CYE, cyanidin chloride equivalents, (*n* = 2 × 5 × 1) ± SD. *^c,d^* Total content of phenolics found by HPLC fingerprint (*n* = 3 × 3 × 1, RSD < 5%). Values in parentheses are the contents of individual compounds: PCA, protocatechuic acid; pHBA, *p*-hydroxybenzoic acid; SQ, quercetin 3-*O*-sophoroside; RT, rutin; HY, hyperoside; IQ, isoquercitrin; GS, sexangularetin 3-*O*-glucopyranoside; QU, quercetin.

**Table 5:** Total content of hydroxycinnamic acids in the analysed *Sorbus* dry extracts and fractions *^a^*.

Sample No. /Extract/ Fraction		Chlorogenic acid isomers (%) *^b^*	Other caffeic acid derivatives (%) *^c^*	*p*-Coumaric acid derivatives (%) *^d^*
1.	ME	6.56 *^I^* (CHA: 4.37; NCHA: 1.25; CCHA: 0.94)	0.88 *^G,H^* (CFA: 0.02)	0.43 *^M^*
	DEF	0.26 *^A^* (CHA: 0.26)	2.66 *^M^* (CFA: 0.51)	not detected
	EAF	4.20 *^E,F^* (CHA: 3.15; NCHA: 0.51; CCHA: 0.54)	5.48 *^R^*	not detected
	BF	14.24 *^R^* (CHA: 10.41; NCHA: 1.98; CCHA: 1.85)	0.56 *^D,E,F^*	0.24 *^I^*
	WR	6.00 *^H^* (CHA: 3.57; NCHA: 1.37; CCHA: 1.06)	0.05 *^A^*	0.03 *^A^*
2.	ME	9.73 *^P^* (CHA: 7.52; NCHA: 1.26; CCHA: 0.96)	1.61 *^J^*	0.36 *^L^*
	DEF	0.64 *^A^* (CHA: 0.40; NCHA: 0.15)	6.10 *^S^* (CFA: 1.14)	1.28 *^R^* (*p*CA: 0.46)
	EAF	7.76 *^K^* (CHA: 6.77; NCHA: 0.45; CCHA: 0.53)	11.07 *^U^*	2.07 *^W^*
	BF	23.83 *^U^* (CHA: 19.49; NCHA: 2.32; CCHA: 2.02)	2.84 *^M^*	1.27 *^R^*
	WR	7.85 *^K,L^* (CHA: 5.46; NCHA: 1.36; CCHA: 1.03)	0.46 *^C,D,E^*	0.10 *^C,D^*
3.	ME	9.98 *^P^* (CHA: 6.80; NCHA: 1.83; CCHA: 1.35)	0.75 *^F,G^*	0.12 *^D,E^*
	DEF	0.69 *^A,B^* (CHA: 0.50; NCHA: 0.19)	4.32 *^Q^* (CFA: 0.48)	0.15 *^E,F^*
	EAF	7.09 *^J^* (CHA: 5.95; NCHA: 0.50; CCHA: 0.63)	7.31 *^T^*	1.43 *^S^*
	BF	16.46 *^T^* (CHA: 12.82; NCHA: 1.93; CCHA: 1.70)	0.66 *^E,F,G^*	0.40 *^L^*
	WR	9.20 *^O^* (CHA: 5.51; NCHA: 2.20; CCHA: 1.50)	0.14 *^A,B^*	0.05 *^A,B^*
4.	ME	6.69 *^I,J^* (CHA: 5.45; NCHA: 0.47; CCHA: 0.76)	0.59 *^D,E,F^*	0.37 *^L^*
	DEF	0.48 *^A^* (CHA: 0.33)	2.65 *^M^* (CFA: 0.41)	0.92 *^P^* (*p*CA: 0.50)
	EAF	8.09 *^K,L,M^* (CHA: 7.06; NCHA: 0.29; CCHA: 0.74)	3.76 *^O^*	1.90 *^U^*
	BF	15.48 *^S^* (CHA: 12.72; NCHA: 0.86; CCHA: 1.90)	1.22 *^I^*	0.99 *^Q^*
	WR	5.39 *^G^* (CHA: 4.13; NCHA: 0.56; CCHA: 0.69)	0.40 *^C,D^*	0.12 *^D,E^*
5.	ME	2.32 *^D^* (CHA: 2.04; NCHA: 0.12; CCHA: 0.16)	1.14 *^I^*	0.21 *^H,I^*
	DEF	0.27 *^A^* (CHA: 0.12)	1.24 *^I^* (CFA: 0.15)	0.91 *^P^* (*p*CA: 0.22)
	EAF	1.16 *^B,C^* (CHA: 1.16)	1.58 *^J^*	0.89 *^P^*
	BF	3.99 *^E^* (CHA: 3.66; CCHA: 0.33)	1.12 *^H,I^*	0.30 *^J,K^*
	WR	2.28 *^D^* (CHA: 1.88; NCHA: 0.16; CCHA: 0.24)	0.75 *^F,G^*	0.03 *^A^*
6.	ME	8.72 *^N,O^* (CHA: 3.72; NCHA: 3.05; CCHA: 1.96)	0.62 *^D,E,F^*	0.19 *^G,H^*
	DEF	0.65 *^A^* (CHA: 0.34; NCHA: 0.19; CCHA: 0.12)	6.10 *^S^* (CFA: 0.27)	0.91 *^P^* (*p*CA: 0.35)
	EAF	5.36 *^G^* (CHA: 3.69; NCHA: 0.77; CCHA: 0.91)	7.10 *^T^*	0.79 *^O^*
	BF	12.78 *^Q^* (CHA: 7.29; NCHA: 3.00; CCHA: 2.49)	not detected	0.33 *^K^*
	WR	8.26 *^L,M,N^* (CHA: 2.78; NCHA: 3.47; CCHA: 2.01)	not detected	not detected
7.	ME	5.05 *^G^* (CHA: 3.99; NCHA: 0.56; CCHA: 0.50)	1.97 *^K^*	0.09 *^C,D^*
	DEF	0.45 *^A^* (CHA: 0.22)	2.25 *^L^* (CFA: 0.45)	0.78 *^O^* (*p*CA: 0.57)
	EAF	1.47 *^C^* (CHA: 1.25; CCHA: 0.21)	11.58 *^W^*	0.38 *^L^*
	BF	8.47 *^M,N^* (CHA: 6.99; NCHA: 0.61; CCHA: 0.87)	1.10 *^H,I^*	0.29 *^J,K^*
	WR	4.53 *^F^* (CHA: 3.30; NCHA: 0.63; CCHA: 0.60)	not detected	not detected
8.	ME	6.54 *^I^* (CHA: 5.86; NCHA: 0.42; CCHA: 0.26)	0.60 *^D,E,F^*	0.17 *^F,G^* (*p*CA: 0.05)
	DEF	0.37 *^A^* (CHA: 0.27)	3.37 *^N^* (CFA: 0.53)	1.50 *^T^* (*p*CA: 1.31)
	EAF	4.07 *^E,F^* (CHA: 3.80; CCHA: 0.26)	4.03 *^P^*	0.52 *^N^*
	BF	10.00 *^P^* (CHA: 8.94; NCHA: 0.60; CCHA: 0.46)	0.32 *^B,C^*	0.28 *^J^*
	WR	6.58 *^I^* (CHA: 5.67; NCHA: 0.47; CCHA: 0.44)	0.44 *^C,D,E^*	0.07 *^B,C^*

^a^ Results are mean values of replicate analyses (*n* = 3 × 3 × 1, RSD < 5%) calculated per dry weight of the extract or fraction. Different superscripts (capitals) in each column indicate significant differences in the mean values at *p* < 0.05. Codification of the samples, extracts and fractions is given in Table 1. ^b,c,d^ Total content of phenolics found by HPLC fingerprint. Values in parentheses are the contents of individual compounds: CHA, chlorogenic acid; NCHA, neochlorogenic acid; CCHA, cryptochlorogenic acid; CFA, caffeic acid; *p*CA, *p*-coumaric acid.

**Figure 3:**
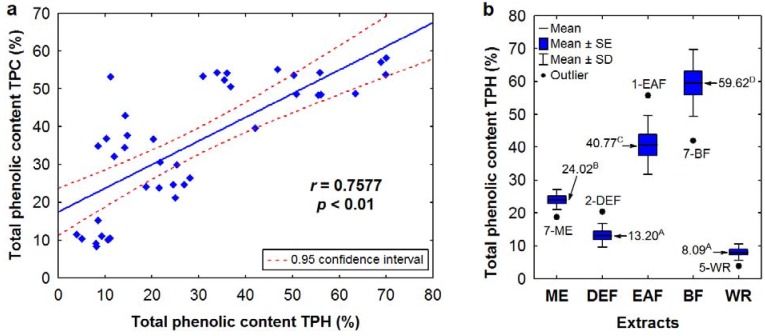
(**a**) Scatter diagram of the correlation between TPC and TPH levels of the *Sorbus* dry extracts. (**b**) Variation in TPH levels among the *Sorbus* extracts depending on the extraction solvent. Sample codes and abbreviations are given acc. to Table 2 and Figure 1. Values marked with different superscript letters are significantlydifferent (*p* < 0.01).

For the majority of extracts, the total phenolic content TPH, calculated as the sum of total proanthocyanidins and individual compounds quantified by HPLC, is consisted with the TPC levels expressed in GAE, which is evidenced by a high and statistically significant correlation between these parameters (Figure 3a). Remarkable differences in these contents were observed only for DEFs, especially for leaf samples, in which the TPC values were 3–5 times higher than the TPH levels. If the DEFs were excluded, the correlation between TPC and TPH levels was stronger (*r* = 0.8859, *p <* 0.01).

As shown in Figure 3b, the highest TPH levels were found for BFs (50.85–69.93% dw) with one outlier found for BF of the *S. pogonopetala* leaf (42.08%) and EAFs (30.89–50.32% dw) with one outlier found for EAF of the *S. aucuparia* inflorescence (55.82%). Since the same extracts were the most active SET-type antioxidants, a high and statistically significant (*p* < 0.01) linear correlation was observed between the TPH contents and the EC_50_ values of the DPPH test (*r* = −0.7411), TEAC (*r* = 0.8019) and FRAP (*r* = 0.6465) values, and this is clear evidence that phenolic compounds are the most important determinants of the SET-type antioxidant activity of the tested extracts. Elimination of DEFs from the correlation test resulted in increased *r* values (−0.8888, 0.8646, and 0.8008 for the DPPH, TEAC and FRAP tests), which suggested that some non-phenolic compounds could synergistically act as antioxidants in DEFs, or that the phenolics present in these extracts exhibit higher antioxidant capacity in comparison to the constituents of other extract types.

**Figure 4:**
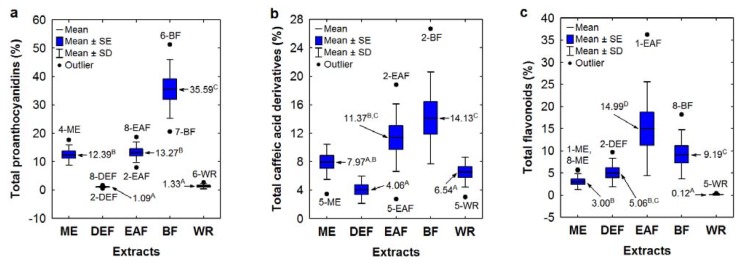
(**a**–**c**) Variation in the levels of main phenolic groups among the *Sorbus* extracts depending on the type of extraction solvent. Sample codes and abbreviations are given acc. to Table 2 and Figure 1. Values marked with different superscript letters are significantly different (*p* < 0.01).

Impact of the individual phenolic groups on SET-type activity of the *Sorbus* extracts was studied by multiple linear regression analysis. Apart from hydroxybenzoic acid derivatives, all other analyte groups (proanthocyanidins, flavonoids and hydroxycinnamic acid derivatives) exhibited significant (*p* < 0.01) partial correlations with the activity parameters. The strongest partial correlations were found between proanthocyanidins and the TEAC values (*r* = 0.6824), between flavonoids and the DPPH EC_50_ (*r* = 0.6086) and FRAP (*r* = 0.6612) values, between *p*-coumaric acid derivatives and the FRAP values (*r* = 0.6058), and between total caffeic acid derivatives (including chlorogenic acid isomers) and the TEAC values (*r* = 0.4789). Since the levels of *p*-coumaric acid derivatives were low (0.00–2.07% dw), three other listed groups of phenolics could be deemed determinants of the tested activity. The levels of these analytes in the extracts were affected mainly by the extracting solvents as shown in the boxplots (Figure 4a–c). The highest levels of total proanthocyanidins were found for BFs (26.51–46.00% dw, without outliers), the highest total content of caffeic acid derivatives were observed for BFs and EAFs (5.10–17.12% dw, without outliers), and the highest total flavonoid levels were found for EAFs (6.61–21.21% dw, without the outlier), which reconfirmed that ethyl acetate and *n*-butanol are the best extractants of *Sorbus* antioxidants.

**Figure 5:**
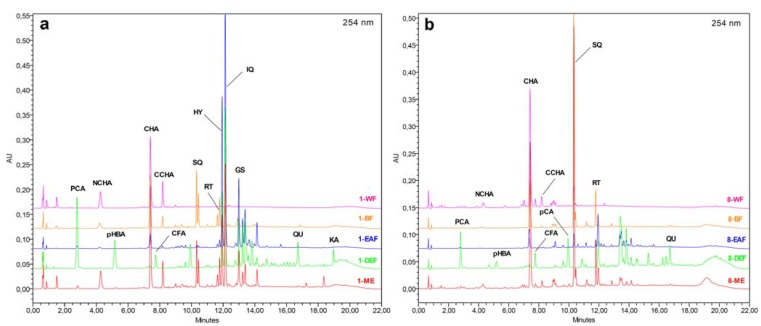
.(**a**–**b**) Representative HPLC fingerprint chromatograms of the *Sorbus* extracts. Sample and peak codes are given acc. to Table 2, Table 4 and Table 5.

In the present work, the first time HPLC fingerprint analysis was performed for the inflorescence and leaf extracts of the tested *Sorbus* species. The individual phenolic acids and flavonoids were identified by comparison of their chromatographic behaviour and PDA spectra with authentic standards, including a set of compounds isolated previously from *Sorbus* plants [23]. Apart from the fully characterised ones, several peaks were tentatively identified and classified into the appropriate groups of phenolics by their PDA spectra, which enabled quantitation of ca. 95% of the UV-absorbing constituents of the extracts. The qualitative phenolic profiles of the tested *Sorbus* species appeared to be similar, and the most important interspecific differences were in quantitative levels of the individual analytes, which was exemplified for the *S. aucuparia* inflorescence (Figure 5a) and *S. wilfordii* leaf (Figure 5b). For the majority of extracts, the dominant components were identified with the standards, but in the case of DEFs of the *S. commixta* inflorescence and the leaves of *S. gracilis* and *S. pogonopetala*, the main constituents could be only tentatively characterised and further isolation and spectroscopic studies are needed for their full structural identification.

The main components of MEs were caffeoylquinic acids (2.32–9.98% dw) and flavonoids (1.30–5.71% dw). The dominant caffeoylquinic acid in all samples was CHA, except the extract of *S. koehneana* inflorescence that contained considerable levels of neo- and cryptochlorogenic acids (NCHA/CCHA). The flavonoid fractions of MEs were abundant in quercetin glycosides, such as quercetin 3-*O*-sophoroside (SQ), RT, hyperoside (HY), isoquercitrin (IQ), and sexangularetin 3-*O*-*β*-D-glucopyranoside (GS, found only in the extracts of inflorescences). Fractionation of MEs between solvents of different polarity yielded fractions of strongly different composition. Simple phenolic acids, such as CFA, *p*-hydroxybenzoic (*p*HBA), *p*-coumaric (*p*CA), and protocatechuic (PCA) acids, as well as flavonoid aglycones, such as QU and kaempferol (KA) were found only in DEFs. On the other hand, flavonoid diglycosides (SQ, RT) were present almost exclusively in BFs (traces of these compounds could be detected in some EAFs and WRs). Flavonoid monoglycosides (HY, IQ, and GS) were found in DEFs, EAFs, and BFs, with the highest levels observed for EAFs. Caffeoylquinic acids were recorded as the major components of BFs and WRs.

Some of the analysed *Sorbus* extracts turned out to be abundant in the individual phenolic metabolites, e.g., BF of the *S. commixta* inflorescence in CHA (19.49% dw), EAF of the *S. aucuparia* inflorescence in IQ (16.15% dw) and HY (9.05% dw), EAF of the *S. decora* inflorescence in HY (10.67% dw), and BF of the *S. wilfordii* leaf in SQ (13.37% dw). Apart from the strong antioxidant activity [11,16,17], these compounds exhibit many other kinds of biological activities, including cholagogic, hypoglycaemic, hypotensive, anti-inflammatory, vaso- and hepatoprotective effects. This activity is a scientific basis of the use of IQ, HY, and CHA as functional food and cosmetic additives [24]. There are, however, only a few plant extracts that accumulate high levels of these phenolics, and the richest are the extracts of green coffee beans (ca. 20% of CHA [25]), *Equisetum arvense* stems (15–38% of IQ [26]), and *Hypericum perforatum* herb (4–19% of HY [27]). The *Sorbus* extracts could thus serve as efficient source materials for isolation of pure compounds.

### 2.4. HAT-Type Antioxidant Activity of the Sorbus Dry Extracts

The ability of *Sorbus* extracts to react *via* the hydrogen atom transfer (HAT) mechanism was screened by testing the inhibition of linoleic acid (LA) peroxidation [28]. In this test, LA was oxidised in a chain reaction initiated by peroxy radicals generated through thermal decomposition of AAPH. This chain reaction can be retarded by an antioxidant donor of H-atom, which scavenges the chain-carrying peroxy radical [29]. The degree of oxidation (level of developed lipid peroxides) was measured using the ferric thiocyanate method [30]. The chain-breaking antioxidant activity of the analytes was expressed as the percentage inhibition of LA-oxidation and was characterised by the IC_50_ value. Although differences in SET-type antioxidant activity between the dry extracts derived from various *Sorbus* tissues were relatively low, they were most pronounced for the extracts of *S. aucuparia* inflorescence and *S. wilfordii* leaf, thus these materials were selected for LA-peroxidation test. As shown in Table 5, the tested extracts exhibited extremely different activity, and these differences were primarily affected by the plant species investigated.

**Table 6:** Antioxidant activity of the selected *Sorbus* dry extracts and fractions in HAT-type test of linoleic acid (LA) peroxidation *^a^*.

Extract/ Fraction	Inflorescence of *S. aucuparia*	Leaf of *S. wilfordii*
	IC_50_ (µg/mL) *^b^*	IC_50 _(µg/mL) *^b^*
ME	112.28 ± 3.37 *^C^*	38.55 ± 1.92 *^A^*
DEF	119.94 ± 5.31 *^C^*	82.21 ± 1.72 *^B^*
EAF	78.14 ± 2.17 *^B^*	78.94 ± 2.34 *^B^*
BF	131.28 ± 3.28 *^D^*	40.12 ± 1.09 *^A^*
WR	228.31 ± 4.11 *^E^*	36.90 ± 1.48 *^A^*

*^a^* Results are mean values of triplicate analyses ± SD calculated per dry weight of the extract or fraction. Different superscripts (capitals) indicate significant differences in the mean values at *p* < 0.01. Codification of the extracts and fractions is given in Table 1. *^b^* IC_50_, inhibition concentration, amount of antioxidant needed to decrease the LA peroxidation by 50%.

The highest activity was found for ME and WR of *S.* *wilfordii* leaf, which was very surprising considering low total phenolic levels and low SET-type activity found for these extracts. There was also no clear correlation between IC_50_ values of LA-peroxidation test and the TPC (*r* = −0.3652), and TPH levels (*r* = −0.2103). However, if the correlation was analysed separately for each of the plants, slight linear dependences could be observed. Although not statistically significant (*p* > 0.05), the negative correlations observed for *S.* *aucuparia* dry extracts (*r* = −0.7750 and −0.5853, respectively) were similar to the results found previously for *in situ* methanolic liquid extracts from several *Sorbus* tissues [8], which reconfirmed the conclusion that phenolics are the main determinants of the HAT-activity of *S.* *aucuparia* extracts. In the case of *S.* *wilfordii*, the absolute value of the coefficient *r* for the relationship with the TPC levels was similar, but the correlation was positive (*r* = 0.7634), which indicated strong differences in the chemistry of antioxidants existing in both plant tissues on the one hand, and that phenolics are not primarily responsible for the HAT-activity of the *S.* *wilfordii* dry extracts on the other hand. The latter conclusion was affirmed by the lack of correlation between the IC_50_ and TPH values (*r* = −0.1869) for this plant. Since the IC_50_ values for DEFs and EAFs were quite similar for both plants, these discrepancies could be affected by extremely polar, non-phenolic constituents of ME of *S. wilfordii* leaf, which are not extractable by diethyl ether and ethyl acetate. The chemical nature of these compounds and their presence in other *Sorbus* species should be strongly addressed for future research.

A critical impact of chemical structure on different reactivity of analytes in SET and HAT reactions was observed also for the standards, as evidenced by low and not-significant (*p* > 0.05) correlation between the results of LA-peroxidation and SET-type tests, *i.e.*, the DPPH (*r* = −0.1317), TEAC (*r* = −0.1167), and FRAP tests (*r* = −0.1417). The activity order of standards in the LA-peroxidation test was also different than in the SET-reactions (Table 3), e.g., the most active was BHA, which was one of the weakest SET-type antioxidants.

The dry extracts from *S. wilfordii* exhibited very high activity as compared with phenolic standards. The most active ME, BF, and WR have comparable or lower IC_50_ values than CA, CHA, QU, RT, and TBHQ, while the activity of CFA, GA, BHA, BHT, and Trolox^®^ was only twice as high. Although the activity of *S. aucuparia* extracts turned out to be lower, in the case of the most active EAF it was still comparable with the activity of CA, CHA, and RT.

## 3. Experimental

### 3.1. Plant Material

Samples of inflorescences and leaves of the studied *Sorbus* species (Table 1) were collected at the flowering stage (June 2009) and authenticated in the Arboretum (51°49°N, 19°53°E), Forestry Experimental Station of Warsaw University of Life Sciences (SGGW) in Rogów (Poland). Voucher specimens were deposited in the herbarium of the Department of Pharmacognosy, Medical University of Łódź, Poland (the voucher specimen numbers have been given in ref. [10]).

### 3.2. Chemicals and Instrumentation

Chromatographic grade purity reagents and standards, such as 2,2-diphenyl-1-picryl hydrazyl (DPPH); 2,2¢-azobis-(2-amidinopropane) dihydrochloride (AAPH); 2,2¢;-azinobis-(3-ethylbenzothiazo-line-6-sulfonic acid) diammonium salt (ABTS); 2,4,6-tris-(2-pyridyl)-s-triazine (TPTZ); (±)-6-hydroxy-2,2,7,8-tetramethylchroman-2-carboxylic acid (Trolox^®^); (+)-catechin monohydrate; caffeic acid; gallic acid monohydrate; chlorogenic acid hemihydrate; quercetin trihydrate; rutin trihydrate; hyperoside, and linoleic acid were purchased from Sigma-Aldrich (Germany/USA). Analytical grade standards of butylated hydroxyanisole (BHA); 2,6-di-tert-butyl-4-methylphenol (BHT); and *tert*-butylhydrochinon (TBHQ) were from the same supplier. All other chemicals and solvents were of analytical grade and from POCh (Poland). In all analyses redistilled water was used.

Organic solvent extracts were evaporated under reduced pressure using a rotary evaporator Rotavapor^®^ (Büchi, Switzerland). Water fractions were lyophilized using an Alpha 1-2/LD Plus freeze dryer (Christ, Germany). Samples were incubated in a constant temperature using a BD 23 incubator (Binder, Germany). Absorbance was measured using a Lambda 25 spectrophotometer (Perkin-Elmer, USA), in 10 mm quartz cuvettes. HPLC analyses were carried out on a Waters 600E Multisolvent Delivery System (Waters, USA) with a PDA detector (Waters 2998) detector scanning in the wavelength range of 220–450 nm; a model 7725 sample injection valve (Rheodyne, CA, USA); a 5 μL injection loop; and a LC workstation equipped with Waters Empower 2 software for data collection and acquisition. A C18 Ascentis^®^ Express column (2.7 μm, 75 mm × 4.6 mm i.d.; Supelco, PA, USA), guarded by a C18 Ascentis^®^ C18 Supelguard guard column (3 μm, 20 mm × 4 mm i.d.; Supelco), was used. Constant temperature of the column was maintained using a Peltier Jetstream Plus 5480 thermostat (Thermotechic Products, Austria). Before injection to HPLC system, samples were filtered through a PTFE syringe filter (13 mm, 0.2 µm, Whatman, USA).

### 3.3. Preparation of Dry Plant Extracts and Fractions

Samples of the plant materials were air-dried under normal conditions, powdered with an electric grinder, and sieved through a 0.315-mm sieve. A portion (40 g) of the pulverised plant material was first extracted with chloroform in a Soxhlet apparatus (500 mL, 48 h), and then refluxed triply for 8 h with 70% (v/v) aqueous methanol (500 mL). The alcoholic extract was evaporated to dryness *in vacuo*, suspended in water and subjected to sequential liquid-liquid extraction with diethyl ether, ethyl acetate and *n*-butanol (8 × 100 mL each). The extracts and fractions were concentrated *in vacuo*, and the water residue was lyophilised. Extraction yield was defined as the amount of dried or lyophilised extract or fraction obtained from 100 g of the dried plant material.

### 3.2. Determination of Total Phenolic Content (TPC)

The amount of total phenolics was determined according to the Folin-Ciocalteu (FC) method [7] with the use of methanolic solutions of the tested extracts and fractions (120–240 μg/mL). Results were expressed as gallic acid (GAE) equivalents per dry weight of the extract or fraction.

### 3.3. Determination of Total Proanthocyanidin Content

The total proanthocyanidin content was quantified by the modified acid/butanol assay [31] with the use of methanolic solutions of the tested extracts and fractions (0.35–2.85 mg/mL). An aliquot of the analysed solution (0.5 mL) was placed in a screw-cap vial and mixed with *n*-BuOH-35% HCl (95:5, v/v, 3 mL) and 2% (w/v) NH_4_Fe(SO_4_)_2_·12 H_2_O in 2 M HCl (0.1 mL). After 45 min of incubation at 95.0 ± 0.2 °C the vial was cooled to 25 °C, and the absorbance was read at 550 nm *versus* the unheated sample used as the blank. The results were expressed as cyanidin chloride (CYE) equivalents per dry weight of the extract or fraction.

### 3.4. HPLC Fingerprint Analysis of Individual Phenolic Compounds

Samples of the tested extracts and fractions (10–50 mg) were dissolved in 70% (v/v) aqueous methanol (10 mL), filtered through a PTFE syringe filter, and the filtrate was directly injected (5 µL) into the HPLC system. The elution system consisted of solvent A (0.5% water solution of orthophosphoric acid, w/v) and solvent B (MeCN) with the elution profile as follows: 0–1 min, 5% B (v/v); 1–16 min, 5–30% B; 16–17 min, 30–50% B; 17–19 min, 50% B; 19–20 min, 50–5% B; 20–25 min, 5% B (equilibration). All gradients were linear. The flow rate was 1.4 mL/min, and the column was maintained at 30 °C. The phenolic compounds were classified into the appropriate groups by their UV-Vis spectra, and the detection wavelength was set at 245 nm for hydroxybenzoic acids, 310 nm for some hydroxycinnamic acids, 325 nm for caffeic acid derivatives including chlorogenic acid isomers, 350 nm for flavonoid glycosides, and 370 nm for flavonoid aglycones. Identification and peak purity tests were made with an automated match system (Waters Empower 2 PDA software) by the comparison of retention times and UV-Vis spectra with reference compounds. Eleven external standards were used for calibration including caffeic acid (CFA), chlorogenic acid (CHA), *p*-coumaric acid (*p*-CA), protocatechuic acid (PCA), *p*-hydroxybenzoic acid (*p*-HBA), rutin (RT), isoquercitrin (IQ), hyperoside (HY), sexangularetin 3-*O*-*β*-D-glucopyranoside (GS), quercetin (QU), and kaempferol (KA). Moreover, the qualitative standards of quercetin 3-*O*-sophoroside (SQ), neochlorogenic acid (NCHA) and cryptochlorogenic acid (CCHA) were used in identification tests. The tentatively identified peaks were quantified as equivalents of the following standards: hydroxybenzoic acids as PCA, chlorogenic acid isomers as CHA, other hydroxycinnamic acid derivatives as CFA or *p*-CA, depending on their UV-Vis spectra, flavonoid diglycosides (mean flavonoids eluting before RT) as RT, flavonoid monoglycosides (mean flavonoids eluting after RT) as IQ, and flavonoid aglycones as QU.

### 3.5. DPPH Free Radical-Scavenging Test

The scavenging activity was determined based on the method of Brand-Williams, Cuvelier, and Berset [10] with slight modifications. The DPPH working solution (37.5 mg/L, 95 μM) was prepared in methanol and equilibrated every day to the absorbance of the negative control of 0.700 ± 0.030 at 517 nm (measured after 60 min of incubation). The negative control was prepared by mixing the DPPH working solution (2 mL) with methanol (1 mL). Five dilutions of all analytes were prepared in methanol-water (70:30, v/v) in the concentration range of 0.8–45.0 μg/mL, depending on the analyte. An aliquot of the sample (1 mL) was added to the equilibrated DPPH working solution (2 mL) and vigorously shaken. After 60 min of incubation in screw-cap vials at room temperature in the dark, the decrease in the absorbance was measured at 517 nm. The samples (1 mL) diluted with methanol (2 mL) were used as blanks. The concentration of the analyte in the reaction medium (in μg/mL) was plotted against the percentage of remaining DPPH using the DPPH calibration curve, and the original EC_50_ value was calculated. Finally, the normalised value was calculated using the following equation: EC_50_ (normalised) = {EC_50_ (original) × 25 μg/mL}/c_0_, where c_0_ (μg/mL) is the DPPH concentration in the negative control after incubation.

### 3.6. ABTS (TEAC) Free Radical-Scavenging Assay

The antioxidant activity was also determined using the TEAC method [11], with some variations. The working solution of ABTS radical cation was prepared through the reaction between potassium persulphate and ABTS [11], and then equilibrated to the absorbance of the negative control of 0.700 ± 0.030 at 734 nm (measured after 15 min of incubation). The negative control was prepared by mixing equilibrated ABTS solution (2 mL) with methanol (1 mL). The assays were made for the same analyte concentrations as prepared for the DPPH tests. An aliquot of the diluted sample (1 mL) was added to the equilibrated ABTS solution (2 mL), vigorously shaken, incubated 15 min in screwcap vials at room temperature and in the dark, and then the decrease in the absorbance was measured at 734 nm. The samples (1 mL) diluted with methanol (2 mL) were used as blanks. Scavenging percentage (%S) of the ABTS radical cation by the samples was estimated as the percentage decrease of absorbance, as calculated using the formula: %S = 100 × (1 − Asample / Acontrol). The concentration of the analyte in the reaction medium (in μg/mL) was plotted against the scavenging percentage, and the original calibration equation was calculated. The EC50 values were calculated from the calibration curve normalised with the intercept value of 0.700 as shows Figure 2. Finally, the activity of the analyte was expressed in terms of TEAC, Trolox^®^ equivalent antioxidant capacity.

### 3.7. Ferric Reducing Antioxidant Power (FRAP) Assay

The FRAP was determined according to the method of Pulido *et al.* [16], with some variations described previously [7]. Prior to the analysis, the analytes were diluted with methanol to the concentrations of 65–126 μg/mL. The antioxidant activity was expressed in micromoles of ferrous ions produced by 1 g of the dry extract, fraction or standard, which was calculated from the eight-point calibration curve of ferrous sulphate.

### 3.8. Linoleic Acid (LA) Peroxidation Test (Ferric Thiocyanate (FTC) Method)

The ability of the analytes to inhibit AAPH-induced LA-peroxidation was assayed according to the method of Azuma *et al.* [28] with some modifications. Five dilutions of all analytes were prepared in methanol-water (70:30, v/v) in the concentration range of 65–126 μg/mL. An aliquot of the analyte solution (0.30 mL) was placed in a screw-cap vial and mixed with 1.3% (w/v) LA in methanol (1.40 mL), 0.2 M phosphate buffer (pH 7.0, 1.40 mL), and water (0.70 mL). The negative control was prepared using methanol (0.30 mL) instead of the sample. Peroxidation was initiated by the addition of 55.30 mM AAPH solution in phosphate buffer (0.20 mL). The vial was incubated at 50.0 ± 0.1 °C in the dark, sampling being carried out every hour for up to at least 5 h until the absorbance of the control reach the value of 0.500 ± 0.030 at 500 nm. The degree of oxidation was measured in quintuplicate according to the ferric thiocyanate method [30]. The reaction mixture (0.10 mL) was diluted with 75% aqueous (v/v) methanol (9.70 mL) and mixed with 20 mM FeCl_2_ solution in 3.5% (w/w) HCl (0.10 mL) and 10% (w/w) aqueous NH_4_SCN solution (0.10 mL). After precisely 3 min the absorbance was measured at 500 nm *versus* 75% methanol. The inhibition ratio (I%) of the peroxidation process was calculated as follows: I% = 100 × (1 − ΔA_sample_ / ΔA_control_), where ΔA is the difference between the absorbance measured at the end and the start of the test, and the IC_50_ value was calculated from the calibration curve.

### 3.9. Statistical Analysis

The samples of each analyte (extract, fraction or standard) were analysed for LA-peroxidation test in triplicate and data is reported as mean (*n* = 3 × 1) ± SD (standard deviation). For other photometric methods two samples of each analyte were assayed, each sample was analysed in quintuplicate and data is reported as mean (*n* = 2 × 5 × 1) ± SD. For HPLC assay three samples of each extract or fraction were analysed in triplicate and data is reported as mean (*n* = 3 × 3 × 1) ± SD. The statistics (calculation of SD, one-way analysis of variance, HSD Tukey’s tests, and linearity studies) were performed using the software StatisticaPl for Windows (StatSoft Inc., Poland).

## 4. Conclusions

The present study demonstrated that the studied *Sorbus* dry extracts possess significant SET-type antioxidant capacity, which strongly correlates with the total phenolic content and depends primarily on the extraction solvent. The best solvents able to concentrate the *Sorbus* antioxidants are *n*-butanol and ethyl acetate. Considering the extraction yield, the use of *n*-butanol is the best for enhancement of the SET-type activity of crude methanolic extracts. In contrast to the SET-type activity, the HAT-reactivity of the extracts appears to depend more strongly on the plant species than on the extraction solvent, and its correlation with the phenolic content is weak. However, a more detailed study using a wider set of *Sorbus* extracts and HAT-type methods is needed to confirm these suggestions. 

Both SET and HAT-type activity of the most potent *Sorbus* extracts is comparable with the activity of several standard antioxidants. Although some of the standards are more active in the particular tests than the *Sorbus* extracts, e.g., gallic acid, caffeic acid and quercetin in the SET-tests, and BHA, BHT, gallic acid and Trolox^®^ in the HAT-test, their excessive use in pure form is burdened with the risk of pro-oxidant and toxic effects. The replacement of these extremely active compounds by plant extracts of milder activity, e.g., *Sorbus* extracts should thus be recommended.

Among the *Sorbus* phenolics, proanthocyanidins, flavonoids and hydroxycinnamic acids were found to be primarily responsible for the tested activity. HPLC-profiling of the extracts led to the identification of chlorogenic acid, isoquercitrin, hyperoside, rutin, and quercetin 3-*O*-sophoroside as the main antioxidant components. Given the extremely high phenolic content, some of the *Sorbus* extracts could serve not only as potent antioxidants for use in food, medicine, cosmetics and other fields that require antioxidants, but also as effective sources for isolation of these analytes.
